# Beyond sodefrin: evidence for a multi-component pheromone system in the model newt *Cynops pyrrhogaster* (Salamandridae)

**DOI:** 10.1038/srep21880

**Published:** 2016-03-03

**Authors:** Ines Van Bocxlaer, Margo Maex, Dag Treer, Sunita Janssenswillen, Rik Janssens, Wim Vandebergh, Paul Proost, Franky Bossuyt

**Affiliations:** 1 Amphibian Evolution Lab, Biology Department, Vrije Universiteit Brussel (VUB), Pleinlaan 2, B-1050 Brussels, Belgium; 2 Laboratory of Molecular Immunology, Department of Microbiology and Immunology, KU Leuven - University of Leuven, Minderbroedersstraat 10 - box 1030, B-3000 Leuven, Belgium

## Abstract

Sodefrin, a decapeptide isolated from the male dorsal gland of the Japanese fire belly newt *Cynops pyrrhogaster*, was the first peptide pheromone identified from a vertebrate. The fire belly salamander and sodefrin have become a model for sex pheromone investigation in aquatically courting salamanders ever since. Subsequent studies in other salamanders identified SPF protein courtship pheromones of around 20 kDa belonging to the same gene-family. Although transcripts of these proteins could be PCR-amplified in *Cynops*, it is currently unknown whether they effectively use full-length SPF pheromones next to sodefrin. Here we combined transcriptomics, proteomics and phylogenetics to investigate SPF pheromone use in *Cynops pyrrhogaster.* Our data show that not sodefrin transcripts, but multiple SPF transcripts make up the majority of the expression profile in the dorsal gland of this newt. Proteome analyses of water in which a male has been courting confirm that this protein blend is effectively secreted and tail-fanned to the female. By combining phylogenetics and expression data, we show that independent evolutionary lineages of these SPF’s were already expressed in ancestral *Cynops* species before the origin of sodefrin. Extant *Cynops* species continue to use this multi-component pheromone system, consisting of various proteins in addition to a lineage-specific peptide.

Sodefrin, from the Japanese word “sodefuri” meaning soliciting, was the first peptide pheromone identified from a vertebrate^1^. This small decapeptide (∼1 kDa) was isolated from the sexually dimorphic dorsal glands of the Japanese fire belly newt *Cynops pyrrhogaster* (Salamandridae). Males of this species use an elaborate courtship display to tail-fan the pheromones produced in these glands via their cloaca to the female (see movie S1). Since the discovery of sodefrin, this decapeptide and its species *Cynops pyrrhogaster* have become a model system for pheromone investigation in aquatically courting salamanders^1^,^2^,^3^,^4^,^5^,^6^,^7^,^8^,^9^,^10^,^11^. Variants of this pheromone were also found in a different population of this species (aonirin) and in its congeneric *C. ensicauda* (silefrin)^7^,^12^. In behavioural tests, these decapeptides were shown to function as female attractants in a species-specific and even population-specific way^3^,^6^,^7^,^12^.

Subsequent studies on chemical communication during courtship in urodelans identified three families of larger, uncleaved proteins as courtship pheromones in lungless salamanders (Plethodontidae). Interestingly, in addition to the family-specific Plethodontid Modulating Factor^13^,^14^,^15^,^16^,^17^ and Plethodontid Receptivity Factor^18^,^19^, these studies revealed protein precursors that are largely similar to sodefrin precursors and were termed Sodefrin Precursor-like Factors^20^ (SPF, Fig. 1). The sodefrin decapeptide and SPF protein pheromones are thus derived from the same gene family, here referred to as the SPF-family (see Fig. 1 for consistency in use of terminology in this paper). Behavioural tests with isolated SPF proteins in both lungless salamanders (Plethodontidae) and newts (Salamandridae) have demonstrated that they function as courtship pheromones that enhance female receptivity^20^,^21^. Recent studies on the expression and evolution of SPF-family transcripts in cloacal glands of Salamandridae showed that, compared to SPF transcripts, sodefrin transcripts contain an extra 62 basepair insertion (Fig. 1, insert indicated in black)^22^. This causes a frameshift in protein translation and generates a sequence (Fig. 1, indicated in dark and light green) from which the decapeptide pheromone can be cleaved (Fig. 1, indicated in dark green). The frameshift originated in an ancestral *Cynops* lineage, indicating that it is not present in other salamanders, and that sodefrin and its variants are likely restricted to the *Cynops* lineage^22^. Because the newly generated sodefrin peptide has no sequence homology with existing salamander SPF protein pheromones, this decapeptide represents an interesting case of the birth of a new pheromone^22^.

The urodelan SPF pheromone family is a complex one in which gene duplications have been very common^22^. The first duplication, already in the Late Palaeozoic, denotes the onset of diversification and gave rise to two SPF lineages that were termed alpha and beta^21^,^22^. Today’s descendant alpha and beta transcripts encode protein pheromones that are still secreted by courting male palmate newts^21^. Although SPF transcripts have been PCR-amplified in the genus *Cynops*^22^,^23^, information on effective translation, precursor abundance, and release of these proteins into the water during courtship is inexistent. As a consequence, it is currently unknown whether *Cynops* species actively use full length SPF pheromones next to sodefrin, or whether the decapeptide has replaced the function of SPF in this genus.

Here we studied the use of SPF proteins during courtship in *Cynops pyrrhogaster*. We first performed RNA-Seq transcriptome analyses of the male pheromone-producing dorsal gland to study the significance of SPF with regard to sodefrin transcript expression in this species. To validate these results, we also performed proteome analyses of water in which a male Japanese fire belly newt had been tail fanning, and demonstrate that multiple SPF proteins are effectively secreted and tail-fanned to the female during courtship (see movie S1). Finally, we combined a phylogeny of SPF-family transcripts with our transcriptome and proteome data to show that both alpha and beta SPF ‘s were already effectively secreted in water before the origin of the peptide sodefrin.

## Results and Discussion

### SPF transcripts are highly expressed in the male dorsal glands

To identify the SPF and sodefrin transcripts in the pheromone-producing dorsal gland of male *Cynops pyrrhogaster*, we performed RACE-PCR with a broad spectrum of primers designed on conserved regions of known SPF-family transcripts (Table S1). Obtained sequences were assembled in 99% contiguous sequences (contigs), which resulted in 25 cDNA sequences. (Table 1, GenBank numbers KU213615-KU213639). Two of these were identified as sodefrin transcripts by the presence of the typical 62 bp nucleotide insert (Table 1, CyPy_SPF_3 and 5), while all others are SPF transcripts. Translated sequences displayed up to 80% pairwise divergence, similar to the diversity found in the palmate newt^21^.

To further investigate the relative expression of sodefrin and SPF transcripts, we performed complete transcriptome sequencing (RNA-seq) of the dorsal gland. This yielded 101.402.858 reads, together constituting 5.2 Giga bases (Gb). Expression analyses resulted in mapping of 17,607,281 of these reads on the transcripts that were obtained by RACE PCR, indicating that at least 17.4% of the total RNA-Seq reads of the dorsal gland belong to the SPF-family. From this subset of reads, 15.2% of the expression could be attributed to the sodefrin transcripts. Investigation of the read mappings showed a relatively uniform distribution (including over the 62 bp insert range) with few aspecific reads, confirming that the expression level of these sodefrin transcripts is a realistic estimate. Uncleaved alpha and beta SPF transcripts accounted for 42.8% and 42% of the SPF-family expression in the dorsal gland, respectively. Our analyses therefore indicate that during the breeding period, SPF transcripts comprise the majority (84.8%) of expression of the SPF-family in the male dorsal gland in *Cynops pyrrhogaster*, indicating that sodefrin transcripts are expressed against this strong background.

### Proteome analyses show high congruence with transcriptome expression data

**Figure 2:**
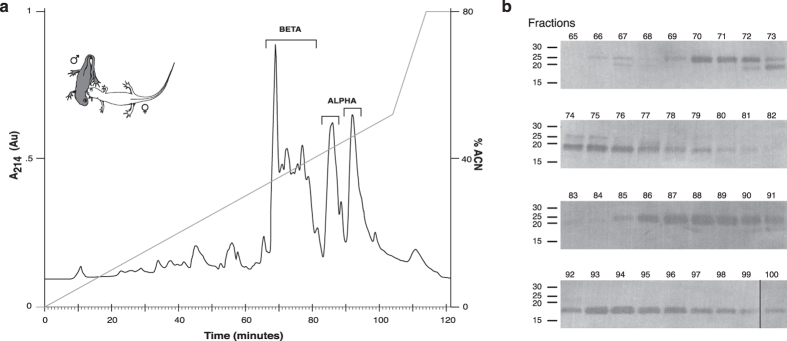
Isolation and identification of SPF proteins secreted during courtship. **(a)** RP-HPLC elution profile of water in which *C. pyrrhogaster* had been courting (drawing top left by Kim Roelants) and **(b)** Coomassie stained blot indicating the presence of multiple SPF proteins in courtship water. Molecular mass markers (in kDa) are indicated on the left and fraction numbers (1 fraction/minute) on top of the blots.

To verify that the RNA-Seq expression data reflect the SPF proteins that are effectively secreted during courtship, we isolated and purified molecules directly from water in which male newts had been courting a female. To account for individual variation, we pooled water of 10 courting couples in which at least five minutes of male tail-fanning occurred. The RP-HPLC chromatogram revealed the presence of several proteins (Fig. 2a) that are absent in water of non-courting couples (see Supplementary Fig. S1). SDS-polyacrylamide gel electrophoresis of the fractions of interest showed the presence of multiple proteins around 20–25 kDa (Fig. 2b), and subsequent Edman degradation of the proteins in these fractions confirmed that they are indeed members of the SPF family (Table S2).

**Figure 3:**
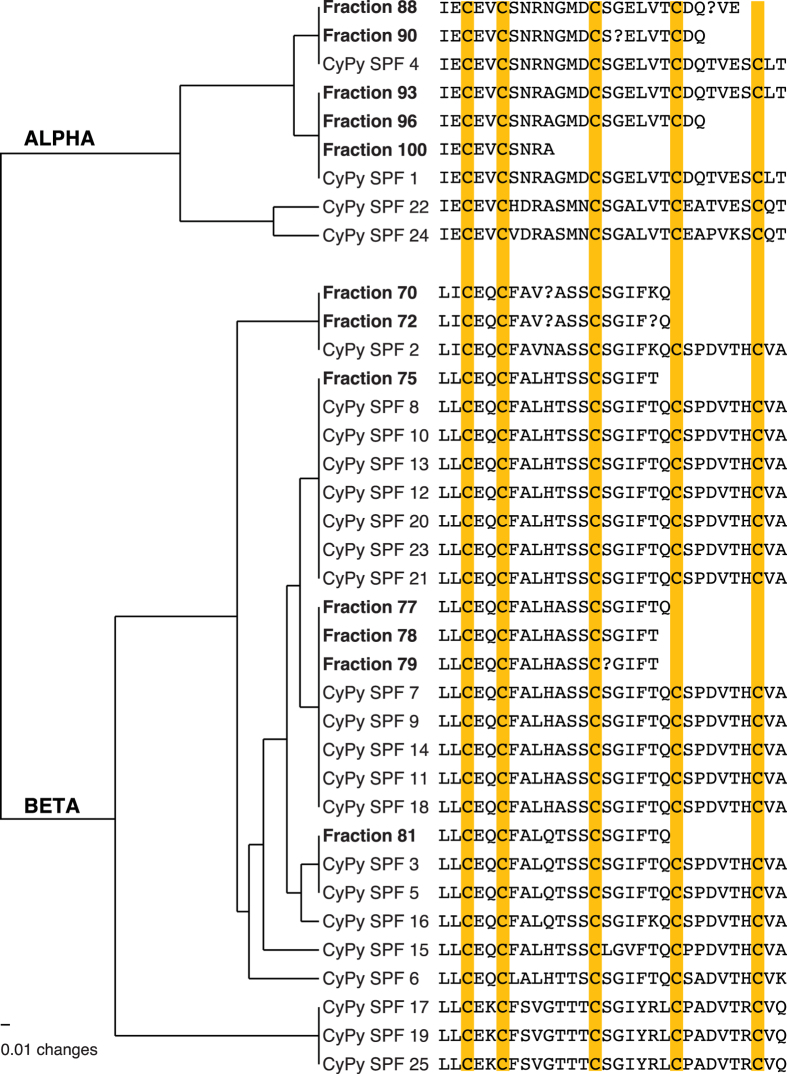
High congruence between transcriptome and proteome expression data. CyPy 1–25 are the SPF-family transcripts found in the dorsal gland of *C. pyrrhogaster*. Transcript names correspond to those in Table 1. Fraction numbers correspond to Fig. 2 and are the SPF proteins that were isolated and Edman sequenced from courtship water. The UPGMA tree with uncorrected distances illustrates a high congruence between expression of SPF transcripts in the dorsal gland and the effectively tail-fanned proteins i.e. the highest expressed SPF transcripts are also retrieved as proteins from courtship water.

The N-terminal sequence already indicates that proteins eluting around minutes 69–81 belong to the beta SPF group, while proteins present in the two peaks around minutes 85–100 belong to the alpha SPF group (Fig. 2a). Mass spectrometry measurements indicate that these proteins are glycosylated (not shown), which is similar to SPF’s in palmate newts^21^, and in line with our *Cynops* precursors showing several potential N-glycosylation sites. To link the proteins from courtship water to our transcripts from the dorsal gland, we aligned a data set combining the amino acid sequences obtained by Edman sequencing (10 to 30 amino acids, see Supplementary Table S2) with our translated transcripts, and estimated the pairwise similarities. The distance tree shows that the Edman sequences correspond to the transcripts that were identified as the most abundant in our RNA expression analyses (Table 1 and Fig. 3).

Since earlier studies (e.g.)^6^, have intensively focussed on recovering cleaved fragments of sodefrin precursors (i.e. sodefrin and peptide variants), our analyses particularly focussed on identifying full-length SPF proteins from courtship water. However, it is noteworthy mentioning that we retrieved a protein (<10 kDa) from courtship water (see Supplementary Table S2, fractions 71–73), which was cleaved from a sodefrin precursor at a lysine-arginine cleavage site. This indicates that, in addition to the decapeptide, other fragments of sodefrin precursors also end up in the water and have the potential of being sensed by a female. Altogether, our data show a high congruence between transcriptome and proteome, indicating that RNA-Seq expression data are a reliable representation of SPF proteins that are effectively tail-fanned from a male to a female during courtship.

### Both alpha and beta SPF’s were expressed at the origin of sodefrin

**Figure 4:**
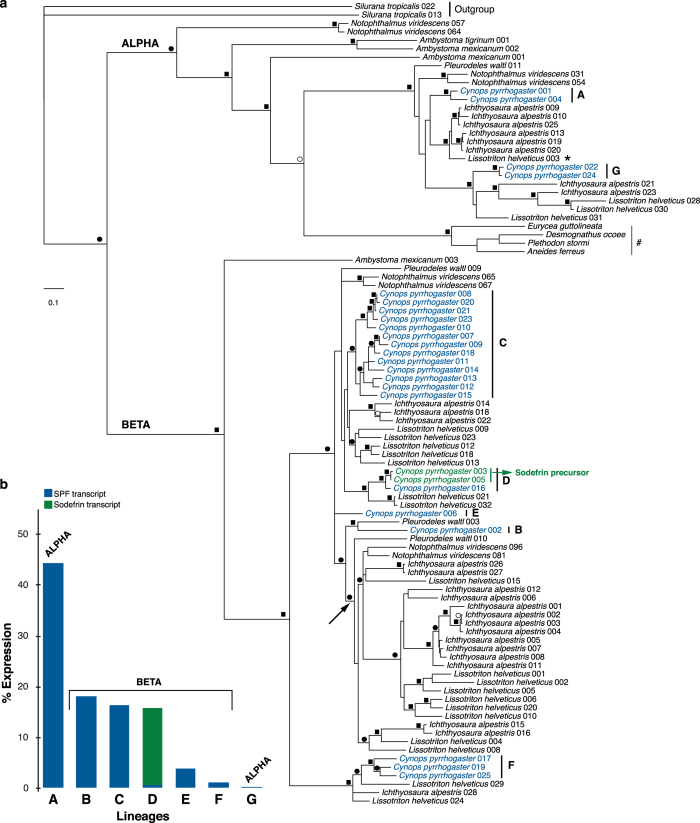
Evolution of precursors in the SPF pheromone family. (**a**) Maximum likelihood tree of translated SPF and sodefrin transcripts after Notung analyses. Squares on the branches indicate Bayesian posterior probabilities (BPP) equal to or higher than 0.95 in combination with ML bootstrap support equal to or higher than 70. Black and white circles indicate support for BPP and ML alone, respectively. (**b**) Comparison of expression (percentage) in the dorsal gland of *C. pyrrhogaster* for seven transcript lineages in the SPF pheromone family.

To investigate the phylogenetic position of SPF-family transcripts of *Cynops* in relation to other known SPF’s, we combined our translated transcripts (full coding sequence) with data from Ambystomatidae^24^,^25^, Plethodontidae^26^ and other Salamandridae^21^,^22^. Alignment resulted in a data matrix of 95 OTUs and 225 characters, for which ProtTest^27^ assigned JTT + G as the best fitting model under the AIC criterion. Subsequent Maximum Likelihood and Bayesian analyses (see Supplementary Fig. S2) in combination with Notung analyses resulted in a gene tree that is highly congruent with previously published relationships among SPF precursor sequences^21^,^22^,^28^ (Fig. 4). The earliest split is formed by the divergence of two supported clades corresponding to alpha and beta SPF protein precursors, whose divergence has been estimated around the Late Paleozoic^21^.

Our tree and associated support values show that currently expressed *C. pyrrhogaster* SPF-family precursors belong to seven independent lineages (Fig. 4: A–G). Six of these lineages exclusively contain SPF precursors (Fig. 4a; A–C,E–G), while a single clade (Fig. 4a; D) combines SPF and sodefrin precursors. Since grouping of any of these lineages would require violating strongly supported nodes in our tree, our analyses indicate that all of these lineages must have originated through gene duplications before the divergence of Asian salamanders (including *Cynops*) from European salamanders (including *Lissotriton* and *Ichthyosaura*)^29^. Because the origin of sodefrin happened in an ancestor of *Cynops* after the divergence of these clades^22^, this indicates that these seven lineages of SPF proteins were existent in *Cynops* at the time of sodefrin origin.

Four lineages currently make up about 95% of the expression of the SPF pheromone family (Fig. 4, A–D). First, the closely related precursors in clade A, together accounting for 44.3% of the expression, belong to a strongly supported alpha clade of SPF proteins (Fig. 4a). These proteins fall in a clade that contains a highly expressed *Lissotriton helveticus* SPF (LiHe 003: see Table S3 and Fig. 4, indicated with *) for which behavioural assays have shown a courtship pheromone function^21^. Furthermore, salamandrid SPF precursors in this clade are orthologous with the main SPF courtship pheromones from the mental gland of plethodontids (Fig. 4, indicated with #)^26^. Altogether, this suggests that alpha SPF’s have continued to play an important role in the courtship behaviour throughout the evolution of internally fertilizing salamanders. Second, the beta-SPF lineages B, C and D account for more than half (50.3%) of the cumulative expression in our RNA-Seq data (Fig. 4), with sodefrin precursors making up the majority of expression in group D (15.2%) (Fig. 4b). In contrast to the alpha precursors, *Lissotriton* orthologs are either absent (lineage B) or only show low expression (lineage C: LiHe 009, 023, 012, 018, 013 lineage D: LiHe 021 & 032) (Fig. 4a; Table S3), while we found no *C. pyrrhogaster* orthologs in the clade that shows the highest expression in *Lissotriton* (Fig. 4a, LiHe 001, 002 and 003, clade of Pleurodelinae indicated with an arrow). Additional BLAST searches for *L. helveticus* orthologs in *de novo* assemblies of our *Cynops* RNA-Seq data only resulted in hits with other SPF precursor clades, confirming that fire belly newts currently make no use of these SPF proteins in courtship. Since the use of multiple individuals for our protein analyses significantly reduces the possibility that the observed expression data are biased by individual variation, our joint analyses suggest that expression shifts between different beta SPF clades have occurred after the divergence of European and Asian newts. In addition to sequence evolution, expression shifts are an alternative way of obtaining species-specificity of pheromone blends in multi-component peptide and protein pheromone systems^30^. Since no expression data are available for newt species that diverged before the *Cynops*-*Lissotriton* split, our data currently do not allow estimating individual transcript abundance in their common ancestor. However, they do support that beta protein pheromones were expressed in the ancestor of *Cynops*, and thus were also present at the timing of the origin of sodefrin. Our combined results thus indicate that *Cynops pyrrhogaster* already effectively secreted both alpha and beta proteins before the origin of sodefrin.

## Conclusion

Despite the fact that sodefrin in the Japanese fire belly newt has served as a model for newt pheromone research since its discovery, the alongside secretion of multiple uncleaved SPF proteins has remained undetected for a long time. Our combined transcriptomic, proteomic and phylogenetic approaches show that this species secretes multiple uncleaved SPF proteins that make up the majority (about 85%) of expression in the SPF pheromone family, and that ancestral forms of both alpha and beta SPF’s were already expressed at the time of sodefrin origin. Because these SPF’s likely continued to perform their pheromone function, the newly originated decapeptide sodefrin could be preserved, even without immediately acquiring a biological function^22^,^31^. Both the place and timing of expression were identical, and the association with functional SPF pheromones likely predetermined sodefrin to develop into a sex pheromone^22^.

Sodefrin and its variants have been shown to be effective as attractants, but since these molecules are specifically released at close distance during tail-fanning, they are likely to function as courtship pheromones in nature^32^. Furthermore, several other fragments ending up in the water after cleavage from sodefrin precursors, some of which still showing homology with SPF’s, have not been tested in behavioural assays that would allow demonstrating a courtship pheromone role. Such tests will be indispensable for a complete understanding of how this multi-component courtship pheromone system originated and evolved.

## Methods

### Ethics statement

*C. pyrrhogaster* individuals (N = 21) were purchased from a local pet shop (Squama, Herent, Belgium). One male was first anesthetized by immersion in 0.5 g/L buffered MS-222 (Sigma-Aldrich) and then euthanized by decapitation and pithing of the brain and spinal canal. This procedure does not violate any European convention (European Convention for the protection of Vertebrate animals used for experimental and other scientific purposes; CETS #123), Belgian law (Art. 2.6 of the Belgian Law of May 4th 1995), or institutional regulation. This research was approved by the Ethical Committee for Animal Experiments of the Vrije Universiteit Brussel (Project number 14-220-35) and carried out in accordance with these approved guidelines.

### Transcriptome analyses

#### RNA sequencing and transcipt expression

For RNA sequencing (RNA-Seq), the part of the dorsal gland extending into the pleuroperitoneal coelom cavity (also known as abdominal gland) was sampled from a *Cynops pyrroghaster* male in breeding mood. Total RNA was extracted using TRI Reagent (Sigma-Aldrich) and the RNAeasy mini kit (Qiagen). A pair-end cDNA sequencing library (PE50) was created by Baseclear (Leiden, The Netherlands) with Illumina TruSeq RNA Library Preparation Kit and fragments were sequenced on an Illumina HiSeq 2500 instrument. FastQ reads were generated after analyses with Illumina Casava pipline (version 1.8.3), a post-filtering script (Baseclear) and FASTQC quality control tool (version 0.10.0) to remove low quality, PhiX-control and adapter reads. *De novo* transcriptome assembly was performed with Trinity^33^ and CLC Genomics Workbench 6.0.4. Transcript expression levels were estimated by mapping reads to the transcripts obtained by RACE PCR, on the RNA-Seq module of the CLC Genomics Workbench 6.0.4, using a similarity fraction of 0.99 and length fraction of 1.00. To compare expression of *Cynops pyrroghaster* with that of *Lissotriton helveticus*, we re-analysed the full-length transcripts from the dorsal gland of the latter species (data from)^21^ by mapping the RNAseq reads on the transcripts under the same conditions.

#### Rapid amplification of cDNA ends

We used the SMARTer-RACE cDNA amplification kit (Clontech) to create RACE cDNA by reverse transcription of 1 μg total RNA extracted from the dorsal gland tissue. To amplify the full coding sequence of the most abundant SPF isoforms, primers were designed on the 3′ untranslated region of the highly expressed SPF isoforms obtained from the RNA sequencing procedure (see above). In addition, eleven degenerate primers from a previous study^21^ were used to obtain a large diversity of SPF protein sequences (Table S1). PCR products were amplified with FastStart High Fidelity Taq DNA polymerase (Roche) using a wide range of annealing temperatures. The following PCR conditions were used: one initial denaturation for 240 s at 94 °C, followed by 36 cycles with denaturation for 40 s at 94 °C, annealing for 60 s at different temperatures, and elongation for 60 s at 72 °C. To clone these amplification products, we used a pGEM-T Easy cloning vector (Promega). Vectors were transformed into TOP10 Competent Cells (Invitrogen). Colonies were picked randomly and inserts were amplified with Faststart Taq DNA polymerase using the same above-mentioned conditions. Amplification products (96 in total) were purified and sequenced, and sequence editing was performed with CodonCode Aligner version 3.7.1.1. Contigs were constructed with 99% identical bases and translated into amino acid sequences.

### Proteome analyses

#### Collection of proteins from courtship water

Pheromone collection was done by placing a male and female for 1 h in a plastic container (25 × 16 × 14 cm) filled with 600 ml of ultrapure water (Milli Q, Millipore, Billerica, MA). During sampling, couples were monitored for courtship behaviour and the amount of time a male fanned his tail was measured. We retained water of 10 courting couples in which at least five minutes of male tail-fanning occurred and in which the female followed the courting male, indicating the presence of pheromones (henceforth termed courtship water). Pheromones were extracted by applying courtship water of a single couple onto two separate solid phase extraction cartridges (RP-C8 and RP-C18 Sep-Pak plus cartridge, 400 mg sorbent, Waters, Milford, MA, USA) using a vacuum pump. As a negative control, this procedure was repeated for 10 non-courting couples that had spent the same amount of time in water as the 10 courting couples. Proteins were eluted from the cartridges with 90% (v/v) acetonitrile containing 0.1% (v/v) trifluoroacetic acid (TFA). All acetonitrile was evaporated using a SpeedVac concentrator (SCV-100 H, Savant instruments, Farmingdale, NY). After concentration, samples were pooled and subjected to reversed-phase high-performance liquid chromatography (RP-HPLC) for additional purification

#### Purification of proteins from courtship water using RP-HPLC

Peptides and proteins were purified using RP-HPLC. After collecting the molecules, pooled and concentrated samples (see above) were loaded onto a Source 5RPC column (4.6 × 150 mm, GE Healthcare Life Sciences, Uppsala, Sweden) pre-equilibrated with 0.1% (v/v) TFA (A). After loading, the column was washed for 10 minutes at a constant flow rate of 1 ml/min using the same solvent. Proteins were eluted with 80% acetonitrile in 0.1% TFA (B) by applying following gradient: from 0–65% B in 104 minutes and from 65–100% B in 10 minutes, at 1 ml/min. Detection of eluting proteins was performed at a wavelength of 214 nm and the eluate was collected in fractions of 1 ml. Fractions of interest were subjected to non-reducing SDS-PAGE using precast gels (Any kD Mini-PROTEAN TGX, Biorad, Hercules, CA, USA). Proteins were visualized by silver staining (Silverquest Silver Staining kit, Invitrogen, Carlsbad, CA, USA, data not shown).

#### Amino acid sequencing

After SDS-PAGE, proteins were transferred from the gel onto a PVDF membrane by semi-dry blotting (Trans Blot Turbo System, Bio-Rad) and stained with 0.1% Coomassie Brilliant Blue R-250 (Sigma, St. Louis, MO, USA; membrane). Protein bands of interest were excised from the blot for N-terminal sequencing on a 491 Procise cLC protein sequencer (Applied Biosystems, Foster City, CA, USA).

### Phylogenetic reconstructions

We combined translated amino acid sequences of the 25 SPF-family transcripts from *C. pyrroghaster* with translated SPF transcripts from four plethodontid, four ambystomatid and 60 other salamandrid sequences from Genbank (Table S4). Two frog Phospholipase A2 Inhibitor (PLI) sequences were chosen as outgroup (Table S4)^22^. Amino acid sequences were aligned using standard automatic parameters implemented in Mafft v7^34^. ProtTest 2.4 was used to select the best fitting model of amino acid (AA) replacement for this data set according to an Akaike Information Criterion^27^. Phylogenetic relationships were estimated under maximum likelihood (ML) with PAUP* under a JTT + G model (as assigned by ProtTest), and in a Bayesian framework using MrBayes 3.2.2^35^,^36^. Bayesian analyses were conducted using a mixed prior for the AA substitution model and gamma correction for among-site rate heterogeneity. Two runs of four Markov chain Monte Carlo (MCMC) chains each were executed in parallel for 10,000,000 generations, with a sampling interval of 1,000 generations. Convergence of the parallel runs was confirmed by split frequency standard deviations (<0.01) and potential scale reduction factors (approximating 1.0) for all model parameters, as reported by MrBayes. Adequate posterior sampling was verified using Tracer 1.6^37^, by checking if the runs had reached effective sampling sizes > 200 for all model parameters. A Bayesian consensus phylogram and Bayesian posterior probabilities (BPP) were inferred from the last 5,000 sampled trees of both runs. Clade support under ML was assessed by 1,000 replicates of rapid bootstrapping using RAXML 7.0.4^38^ on the CIPRES Science Gateway v3.3^39^. Finally, while constraining clades that were supported by BPP (≥0.95) or high bootstrap values (≥70), speciation-duplication analyses in Notung v2.6^40^ were used to find the phylogenetic tree that minimizes the number of gene duplications and losses, and branch lengths were re-estimated from that tree.

## Additional Information

**How to cite this article**: Van Bocxlaer, I. *et al.* Beyond sodefrin: evidence for a multi-component pheromone system in the model newt *Cynops pyrrhogaster* (Salamandridae). *Sci. Rep.* **6**, 21880; doi: 10.1038/srep21880 (2016).

## Supplementary Material

Supplementary Information

## Figures and Tables

**Figure 1:**
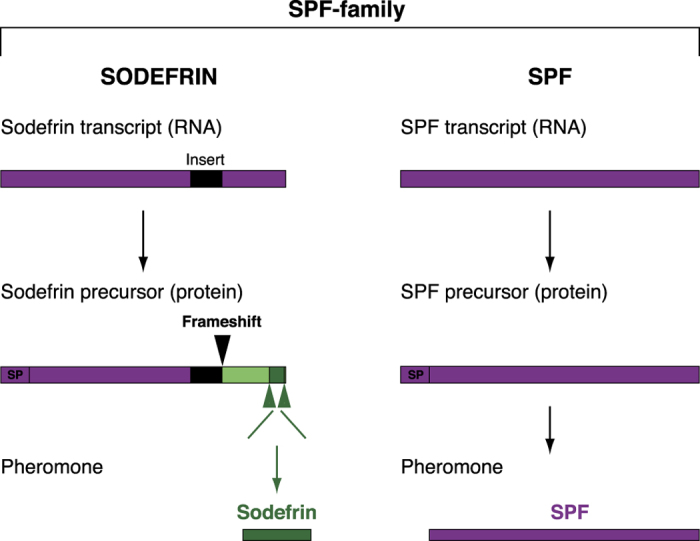
Schematic comparison of sodefrin and SPF. Sodefrin and SPF pheromones belong to the same gene family, here referred to as the SPF (pheromone) family, and are homologues (indicated in purple) at the nucleotide level. The major difference is that sodefrin RNA transcripts contain a 62 base pair insert (indicated in black) that causes a translational frame shift at the C-terminal end of the protein (indicated in light + dark green). This leads to the origin of cleavage sites (indicated with green arrows) that allow generating the decapeptide sodefrin (indicated in dark green). SPF transcripts do not have such an insert, and become expressed as full-length proteins. The pheromones that result from each precursor type therefore show no amino acid sequence similarity. SP = signal peptide.

**Table 1:** SPF-family transcript expression in the dorsal gland of *Cynops pyrrhoghaster*, ranked according to reads per kilobase per million (RPKM) values.

Name	Gene length	RPKM	% RPKM	Unique gene reads	Total gene reads
CyPy_SPF_1	753	458.927	34,41	5.913.474	6.084.698
CyPy_SPF_2	833	241.603	18,11	3.543.589	3.543.634
CyPy_SPF_3	738	142.199	10,66	786.539	1.847.790
CyPy_SPF_4	737	132.387	9,93	1.668.229	1.717.963
CyPy_SPF_5	734	60.250	4,52	344.312	778.674
CyPy_SPF_6	686	52.026	3,90	612.800	628.411
CyPy_SPF_7	662	41.813	3,13	232.970	487.380
CyPy_SPF_8	751	35.040	2,63	190.772	463.342
CyPy_SPF_9	743	32.691	2,45	244.395	427.685
CyPy_SPF_10	706	28.199	2,11	288.031	350.547
CyPy_SPF_11	520	16.174	1,21	127.386	148.092
CyPy_SPF_12	687	13.107	0,98	89.451	158.550
CyPy_SPF_13	718	12.924	0,97	91.112	163.385
CyPy_SPF_14	718	10.545	0,79	108.983	133.310
CyPy_SPF_15	720	9.150	0,69	103.095	115.999
CyPy_SPF_16	661	8.514	0,64	82.660	99.091
CyPy_SPF_17	677	8.237	0,62	83.855	98.193
CyPy_SPF_18	662	7.877	0,59	51.851	91.813
CyPy_SPF_19	673	6.641	0,50	66.292	78.696
CyPy_SPF_20	675	6.320	0,47	37.831	75.111
CyPy_SPF_21	684	3.033	0,23	18.493	36.528
CyPy_SPF_22	757	2.776	0,21	23.431	37.005
CyPy_SPF_23	668	1.467	0,11	7.402	17.255
CyPy_SPF_24	770	1.088	0,08	9.425	14.745
CyPy_SPF_25	685	778	0,06	4.664	9.384

Transcript 3 and 5 are sodefrin transcripts, i.e. transcripts that contain an insertion leading to a frame shift from which a peptide pheromone can be cleaved, while all remaining are SPF transcripts.
