# Supplementary material for: Beyond sodefrin: evidence for a multi-component pheromone system in the model newt Cynops pyrrhogaster (Salamandridae)
Source: Sci Rep. 2016 Mar 3;6:21880. doi: 10.1038/srep21880 (PMC4776240; doi:10.1038/srep21880)
Supplement: Supplementary Information [file srep21880-s1.pdf]

Supplementary information

Beyond sodefrin: evidence for a multi-component pheromone system in the model newt *Cynops pyrrhogaster* (Salamandridae)

Ines Van Bocxlaer[1], Margo Maex[1], Dag Treer[1], Sunita Janssenswillen[1], Rik Janssens[2], Wim Vandebergh[1], Paul Proost[2] and Franky Bossuyt[1]

1 Amphibian Evolution Lab, Biology Department, Vrije Universiteit Brussel (VUB), Pleinlaan 2, B-1050 Brussels, Belgium

2 Laboratory of Molecular Immunology, Department of Microbiology and Immunology, Laboratory of Molecular Immunology, KU Leuven - University of Leuven, Minderbroedersstraat 10 - box 1030, B-3000 Leuven, Belgium

Contains:

Movie S1: Courtship display in *Cynops pyrrhogaster*.

Figure S1: RP-HPLC elution profile of non-courting male female water.

Figure S2: Maximum Likelihood tree of SPF precursors.

Table S1: Primers used in this study.

Table S2: Edman sequences of proteins in courtship water.

Table S3: Estimated expression of SPF precursors in the dorsal gland of *L. helveticus*

Table S4: Accession numbers of species used in the phylogenetic analyses.

Movie S1: Courtship display in *Cynops pyrrhogaster*.

http://dx.doi.org/10.6084/m9.figshare.1619847

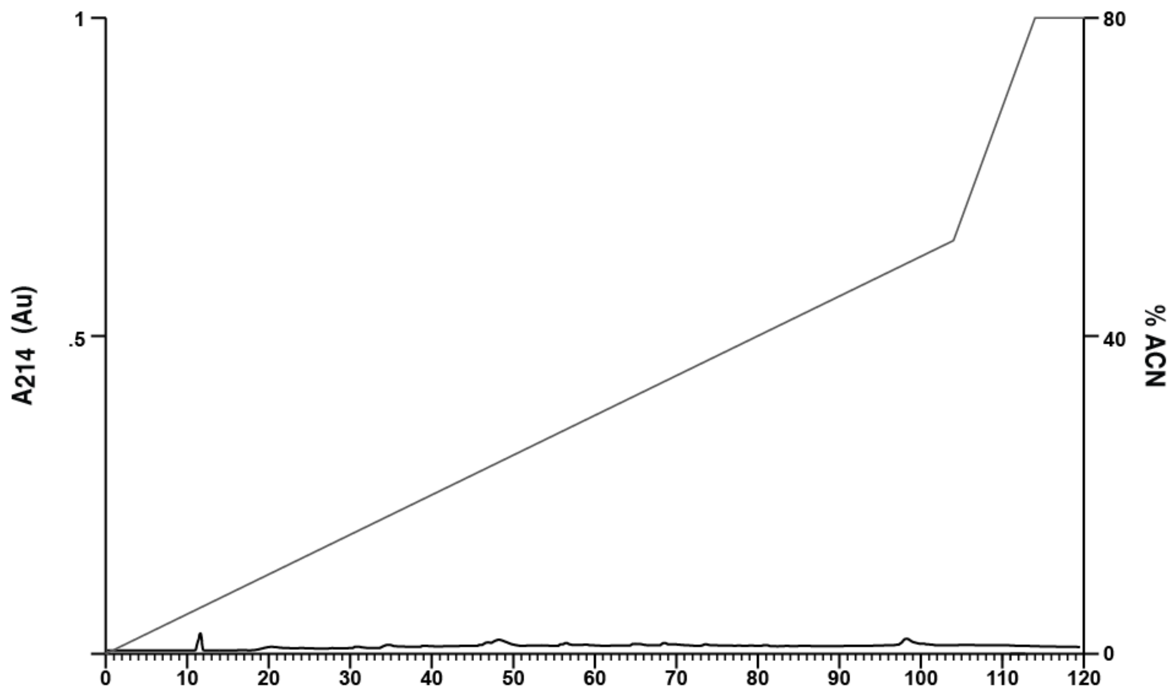

Figure S2: RP-HPLC elution profile of non-courting male female water.

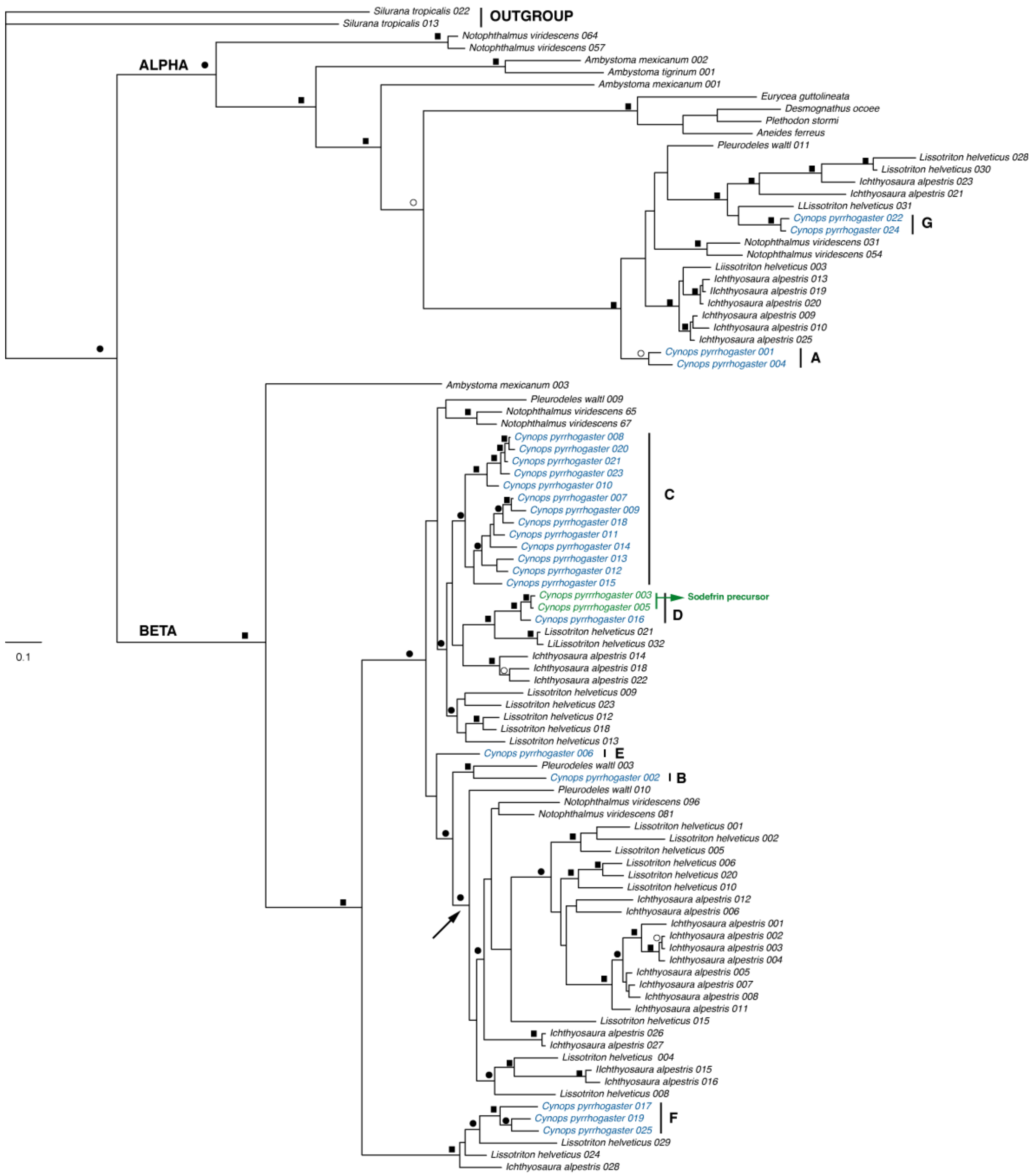

Figure S2: Maximum Likelihood tree of SPF precursors. Squares on the branches indicate Bayesian posterior probabilities (BPP) equal to or higher than 0.95 in combination with ML bootstrap support equal to or higher than 70. Black and white circles indicate support for BPP and ML alone, respectively.

Table S1: primers used in this study

Name	Sequence (5’ – 3’)	Study
SPF43	TTGTTAATAAWYATTCTGTAAAGARGCT	[1]
SPF44	GCCTTGTTGBCAAAAHKTCTTC	
SPF45	ACAAYTWCTAAGCTGGHKTAGGA	
SPF46	GTGTGTATWTGRGGTATRAACAAAGGTC	
SPF47	CCAACAATTACTRRGMKGGAGTAGG	
SPF48	CAACTACTAAGCTRRAGTMRGAGTGC	
SPF49	GGRTAGGATTGCGTCAGATGTT	
SPF50	TAGGAATGTTTCTAYKGACKACTACTRAG	
SPF51	CTATTGCTAAGCTGKGGTG	
SPF52	CAAGWYCAGACTCTGCATGCCA	
SPF53	CACTGCAKTCCACATWCTTGTA	
SPF54	GCTGGCACATGGGCATGT	
SPF55	GCCCAWACASKACTAAGCACATT	
SPF56	GACTCTGVATTHCAGGTACTTGTAGAG	
SPF57	GCACACAGATCATACATTGTTTTTA	This study
SPF58	AGCACACAGATCATACATTGTTTG	
SPF59	GCACACAGATCATACATTGTTGG	
SPF60	CATTCATCAGCTTCAATCTTACTATAG	
SPF61	CATTCATCAGCTTCAATCTTACTATGT	
SPF62	CATTCATCAGCTTCAATCTTACTG	
SPF63	CTATGGGGTATGAACAAAAGTCTCAAAC	
SPF64	CTATGGGGTATGAACAAAGGTCTCAAAG	
SPF65	ATCAAGGTACATAGATGTGGAGTT	
SPF66	CAAGGTACATAGATGTGGAGCC	
SPF67	AAGGTACATAGATGAGGAGTCATA	

Table S2: Edman sequences of proteins in courtship water. Fraction numbers and gel correspond to Figure 2. The bands <10 kDa are not shown. Cysteines were not detected, as they were not alkylated prior to the sequence analysis. Small letters indicate uncertain signals.

Fraction N°	Sequence	Gel
70	LIXEQXFAVXASSxSGIfKQ	
72	LIXEQXFAVXASSxSGIFXQ	Upper band
73	LIXEQXFAVHTSSxsgIFTQ<br>L LN	Lower band
75	LLXEQXFALHTSSxSGIFT	Lower band
77	LLXEQXFALHASSxSGIFTq<br>i d	
78	LLXEQXFALHASSxSGIFT	
79	LLXEQXFALHASSxXGIFT	
81	LLXEQXFALQTSSxSgIFTq	
88	IEXEVXSNRNGMDXSGELVTXdQxve	
90	IEXEVXSNRNGMDXSXELVTXdQ	
93	IEXEVXSNRAGMDXSGELVTXDQTVESXLT	
96	IEXEVXSNRAGMDXSGELVTXDQ<br>T	
100	IEXEVXSNRA	
71-73	LFWSYFPIrKXhVA	<10 kDa

Table S3: Estimated expression of SPF precursors in the dorsal gland of *Lissotriton helveticus*, ranked on RPKM value. The number of reads analysed was 104.081.336, the number of reads that mapped on our SPF/sodefrin precursors was 7.157.215 (6,88%).

Name	Gene length	RPKM	% RPKM	Unique gene reads	Total gene reads
LiHe_SPF_1	701	467.280	33,23	2.318.554	2.344.440
LiHe_SPF_2	724	425.711	30,27	2.184.949	2.205.960
LiHe_SPF_3	696	208.004	14,79	1.036.151	1.036.154
LiHe_SPF_4	738	190.765	13,57	1.007.590	1.007.623
LiHe_SPF_5	704	50.664	3,60	252.932	255.282
LiHe_SPF_6	691	15.722	1,12	77.461	77.757
LiHe_SPF_8	612	13.041	0,93	57.121	57.123
LiHe_SPF_9	705	8.367	0,59	41.941	42.217
LiHe_SPF_10	692	6.195	0,44	30.673	30.681
LiHe_SPF_12	671	4.739	0,34	17.139	22.761
LiHe_SPF_13	703	3.215	0,23	16.171	16.178
LiHe_SPF_21	678	2.740	0,19	9.042	13.294
LiHe_SPF_15	681	2.591	0,18	12.627	12.629
LiHe_SPF_18	683	2.457	0,17	9.449	12.012
LiHe_SPF_20	691	1.536	0,11	7.553	7.598
LiHe_SPF_23	675	1.048	0,07	4.786	5.063
LiHe_SPF_24	687	794	0,06	3.870	3.902
LiHe_SPF_32	678	752	0,05	2.420	3.648
LiHe_SPF_30	762	272	0,02	751	1.482
LiHe_SPF_29	687	174	0,01	846	857
LiHe_SPF_28	761	84	0,01	244	458
LiHe_SPF_31	760	18	0,00	96	96

Table S4: Accession numbers and tissue origin of SPF precursor sequences of species used in the phylogenetic analyses.

Species	Accession number	Tissue	Database
Lissotriton helveticus 001-006	KJ402326-KJ402331	male abdominal gland	NCBI
Lissotriton helveticus 008-010	KJ402333-KJ402335	male abdominal gland	NCBI
Lissotriton helveticus 012-013	KJ402337-KJ402338	male abdominal gland	NCBI
Lissotriton helveticus 015	KJ402340	male abdominal gland	NCBI
Lissotriton helveticus 018	KJ402343	male abdominal gland	NCBI
Lissotriton helveticus 020-021	KJ402345-KJ402346	male abdominal gland	NCBI
Lissotriton helveticus 023-024	KJ402348-KJ402349	male abdominal gland	NCBI
Lissotriton helveticus 028-032	KJ402353-KJ402357	male abdominal gland	NCBI
Ichthyosaura alpestris 001-016	KP849562-KP849577	male abdominal gland	NCBI
Ichthyosaura alpestris 018-023	KP849579-KP849584	male abdominal gland	NCBI
Ichthyosaura alpestris 025-028	KP849586-KP849589	male abdominal gland	NCBI
Notophthalmus viridescens 031	KP118902	male cloacal tissue	NCBI
Notophthalmus viridescens 054	KP118912	male cloacal tissue	NCBI
Notophthalmus viridescens 057	KP118898	male cloacal tissue	NCBI
Notophthalmus viridescens 064-065	KP118895-KM463868	male cloacal tissue	NCBI
Notophthalmus viridescens 067	KM463870	male cloacal tissue	NCBI
Notophthalmus viridescens 081	KP118927	male cloacal tissue	NCBI
Notophthalmus viridescens 096	KP118917	male cloacal tissue	NCBI
Cynops pyrrhogaster 001-025	KU213615-KU213639	male abdominal gland	NCBI
Pleurodeles waltl 003	KM463924	male cloacal tissue	NCBI
Pleurodeles waltl 009	KM463930	male cloacal tissue	NCBI
Pleurodeles waltl 010	KM463931	male cloacal tissue	NCBI
Pleurodeles waltl 011	KM463932	male cloacal tissue	NCBI
Eurycea guttolineata	AAZ06338	male mental gland	NCBI
Aneides ferreus	AAZ06335	male mental gland	NCBI
Desmognathus ocoee	AAZ06329	male mental gland	NCBI
Plethodon stormi	DQ097067	male mental gland	NCBI
Ambystoma mexicanum 001	CN041146	larval limb tissue	NCBI
Ambystoma mexicanum 002	CN035733	larval limb tissue	NCBI
Ambystoma mexicanum 003	I1698676-GIB5VLK02G78TL	brain, blood, etc.	SGP/Sal-site
Ambystoma tigrinum	CN048649	brain tissue	NCBI
Silurana tropicalis 001	F6PQG9	n.a.	Uniprot
Silurana tropicalis 002	XP_002943341	n.a.	NCBI

References

1. Van Bocxlaer, I. et al. Side-by-side secretion of late Palaeozoic diverged courtship pheromones in an aquatic salamander. *P. Roy. Soc. B-Biol. Sci.* **282**, 20142960 (2015).
